# HEDD: the human epigenetic drug database

**DOI:** 10.1093/database/baw159

**Published:** 2016-12-26

**Authors:** Yunfeng Qi, Dadong Wang, Daying Wang, Taicheng Jin, Liping Yang, Hui Wu, Yaoyao Li, Jing Zhao, Fengping Du, Mingxia Song, Renjun Wang

**Affiliations:** 1 Department of Bioscience, School of Life Science, Jilin Normal University, Siping, China; 2 Department of Computer Science and Technology, Computer College, Jilin Normal University, Siping, China; 3 Department of Social Physical Education, Physical Education College, Jilin Normal University, Siping, China; 4 Department of Biotechnology, School of Life Science, Jilin Normal University, Siping, China

## Abstract

Epigenetic drugs are chemical compounds that target disordered post-translational modification of histone proteins and DNA through enzymes, and the recognition of these changes by adaptor proteins. Epigenetic drug-related experimental data such as gene expression probed by high-throughput sequencing, co-crystal structure probed by X-RAY diffraction and binding constants probed by bio-assay have become widely available. The mining and integration of multiple kinds of data can be beneficial to drug discovery and drug repurposing. HEMD and other epigenetic databases store comprehensively epigenetic data where users can acquire segmental information of epigenetic drugs. However, some data types such as high-throughput datasets are not provide by these databases and they do not support flexible queries for epigenetic drug-related experimental data. Therefore, in reference to HEMD and other epigenetic databases, we developed a relatively comprehensive database for human epigenetic drugs. The human epigenetic drug database (HEDD) focuses on the storage and integration of epigenetic drug datasets obtained from laboratory experiments and manually curated information. The latest release of HEDD incorporates five kinds of datasets: (i) drug, (ii) target, (iii) disease, (vi) high-throughput and (v) complex. In order to facilitate data extraction, flexible search options were built in HEDD, which allowed an unlimited condition query for specific kinds of datasets using drug names, diseases and experiment types.

**Database URL:** http://hedds.org/

## Introduction

Eukaryotic DNA is packaged into chromatin, which incorporate repeating nucleosomes by wrapping 1.67 turns around a histone octamer that comprise of two molecules each of the common histones H2A, H2B, H3 and H4 (1). In the 1970s, it was described that the addition of a methyl group at the fifth position of the cytosine in a CpG dinucleotide could inactivate gene expression. Furthermore, the N-terminal tail of the histone is subject to many chemical modifications such as methylation, acetylation, ubiquitylation, phosphorylation and ADP-ribosylation. The discovery that reversible molecular modifications of DNA and histones control gene expression introduced a novel paradigm known as epigenetics (2–4). Epigenetic enzymes ‘write’ the DNA methylation and histone code and ‘erase’ the histone code. The recognition of these changes by adaptor proteins ‘read’ the histone code (5, 6). Working in concert, three classes of epigenetic proteins (‘writers’, ‘erasers’ and ‘readers’) function to determine whether genes are turned on or off, and the deregulation of these processes plays a central part in several diseases (7).

**Figure 1:**
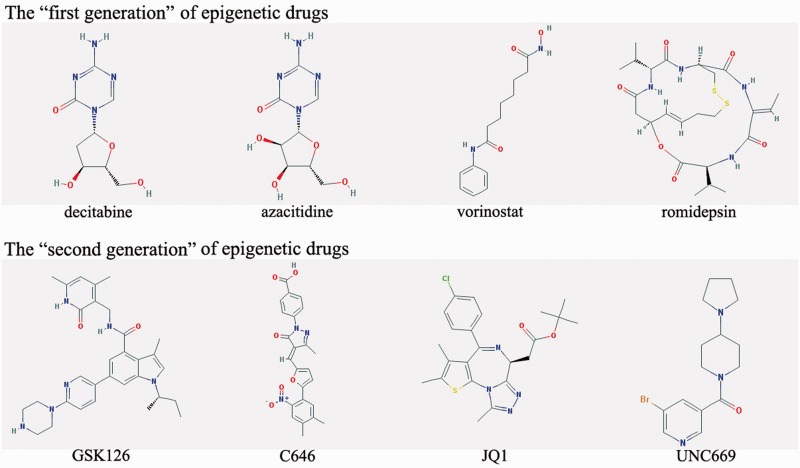
The chemical structures of representative ‘first and second generation’ of epigenetic drugs.

In the past decades, epigenetics has emerged as a novel and important research area in drug discovery, which includes most of the human pathology in which deregulation in gene expression is observed (8). Historically, compounds that demonstrated DNA methyltransferase inhibitor (DNMTi) activity in cells were cytidine analogs decitabine and azacitidine; and these were approved for myelodysplastic syndrome (MDS) treatment (9). Moreover, hydralazine and procainamide have been approved for hypertension and cardiac arrhythmia treatment, respectively. Recently, its activity as DNMTi has been discovered (10). The histone deacetylase inhibitor (HDACi) vorinostat and romidepsin were approved by the FDA (Food and Drug Administration) for cutaneous T-cell lymphoma (CTCL) (8, 11). Panobinostat and belinostat are FDA approved for the treatment of multiple myeloma (MM) and peripheral T-cell lymphoma (PTCL), respectively (12, 13). These compounds are clear examples of the therapeutic relevance of ‘first generation’ of epigenetic drugs for clinical application, which are DNMTis and HDACis (5).Great progress has been made in developing ‘second generation’ of epigenetic drugs, which are small molecule inhibitors of other epigenetic enzymes and adaptor proteins, including histone methyltransferases (HMTs), histone acetyltranferases (HATs), histone demethylases (HDMs), proteins binding to methylated and acetylated histones (PAHs and PMHs). Figure 1 exhibits the chemical structures of representative ‘first and second generation’ of epigenetic drugs. The ‘second generation’ of epigenetic drugs are also entering clinical trials. For example, the HMT inhibitor (HMTi) GSK126 provides preclinical validation of EZH2 (Enhancer of zeste homolog 2) activating mutations as a marker of selectivity for an EZH2 inhibitor in diffuse large B-cell lymphoma (DLBCL) (14). The most potent and selective HAT inhibitor (HATi) C646 is a potent and selective inhibitor of p300, and it can reduce histone acetylation and cancer cell growth (15). The HDM inhibitor (HDMi) EPZ004777 selectively inhibits cell H3K79 methylation and restrains key mixed lineage leukemia (MLL) fusion expression of target genes (16). The PAH inhibitor (PAHi) JQ1 promotes differentiation, tumor regression and prolonged survival in murine models of the NUT (nuclear protein in testis) midline carcinoma (NMC), which is consistent with the role of bromodomain-containing protein 4 (BRD4)-NUT in this rare cancer (17). The PMH inhibitor (PMHi) UNC669 is the first co-crystal structure of a small molecule bound to Lethal(3)malignant brain tumor-like protein 1 (L3MBTL1) (18). The proven clinical utility of DNMTi and HDACi, as well as the rapid preclinical advancement of ‘second generation’ of epigenetic drugs, lends optimism for future epigenetic drug discovery and development.

With the advancement of epigenetic drug researches, laboratories and researchers around the world have profiled a mass of epigenetic drug data (i.e. high-throughput profile, clinical trials, target binding constants, and co-crystal structure of the drug-target complex). As shown in Table 1, by analyzing and applying these data, researchers have demonstrated the action mechanism of epigenetic drugs, and have made great efforts in epigenetic drug discovery and repurposing(19–22). Moreover, the vast number clinical trials underlines the promising use of these drugs in the diagnostic therapeutics of human diseases (23–25), which also provide a large number of guidance and reference for researchers. Furthermore, target binding constants and structures of drugs and the drug-target complex are useful for epigenetic drug optimization and discovery (26, 27).

**Table 1:** Researches related to epigenetic drugs

Drugs	Research contents	References
Vorinostat	Analyzed time-series gene expression data to elucidate vorinostat induced apoptosis	Qi et al. (19)
Azacytidine	Analyzed azacytidine on genome-wide DNA methylation patterns in lung cancer	Hascher et al. (20)
TSA and vorinostat	Measured changes of vascular chromatin modifications treated with TSA and vorinostat	Rafehi et al. (21)
Decitabine	Developed a molecular means at diagnosis for responsive or resistant to decitabine	Meldi et al. (22)
Valproic acid	Conducted clinical trials of valproic acid in treatment for AML	Bouzar et al. (24)
Valproic acid	Conducted clinical trials of valproic acid in treatment for HIV infection	Archin et al. (25)
Azacytidine	Determined the structures of PaPth in its native and bound states with azacytidine	Singh et al. (27)

It is useful to have a repository of these data. This would boost calls for the comprehensive collection and systemization of epigenetic drug-related experimental data in in-depth data mining. There have been few resources for drugs or chemical compounds such as ChEMBL (28), PubChem Compound (29), DrugBank (30) and ZINC (31). These general drug databases contain huge numbers of compounds, and it would be time-consuming for users to search for a drug’s related information. Epigenetic databases HEMD (32) and ChromoHub (33) store multiple kinds of epigenetic data, where users can search for human epigenetic enzymes and chemical modulators and map on phylogenetic trees disease associations, protein structures, chemical inhibitors and histone substrates, respectively. Using them, users can acquire information of a chemical inhibitor by browsing different sections within the webpage of a chemical inhibitor. As these databases are not specially designed for epigenetic drug-related experimental data, some data types are not contained such as high-throughput datasets, cell-type/disease specific with combination therapeutic trials, target binding constants, or co-crystal structure of the drug-target complex. Moreover, they do not support flexible queries for epigenetic drug-related experimental data. In a word, these databases are not customized and integrated for biologists and bioinformaticians who look for new convenient resources of information relating to epigenetic drugs. To the best of our knowledge, there has been no specialized database that focuses on epigenetic drugs in mammals, which hinders further systematic and in-depth data mining. Therefore, there is need to build a database that is dedicated to the storage of epigenetic drug-related experimental data. A database of this kind would be beneficial to epigenetic drug studies such as the identification of a drug target for a specific cell-type/disease, and elucidate the drug activation at an Omics level.

In reference to HEMD and other epigenetic databases, we developed the human epigenetic drug database (HEDD), which is available at http://www.hedds.org. HEDD focuses on the storage and integration of epigenetic drug information obtained from experimental data. The latest release of HEDD provides five kinds of datasets: (i) drug, (ii) target, (iii) disease, (vi) high-throughput and (v) complex. HEDD incorporates a set of tools for querying and browsing different datasets. Furthermore, schematism was adopted to interpret the structure of drug, drug-target complex and the interaction within the binding pocket. By integrating the Jmol Project (https://sourceforge.net/projects/jmol), HEDD provids a visual function to display the 3D structure of epigenetic drugs and the drug-target complex. HEDD may be a useful resource for a variety of biologists, primarily bench scientists, who look for new convenient resources of epigenetic drugs. Moreover, information relating to HEDD is also meritorious for computational biologists for *in silico* approaches, in order to advance epigenetic drugs, mine the epigenetic-relevant chemical space, uncover the structure activity relationship (SAR), and assist computer-aided drug design (CADD).

## Database construction and content

### Data source

**Figure 2:**
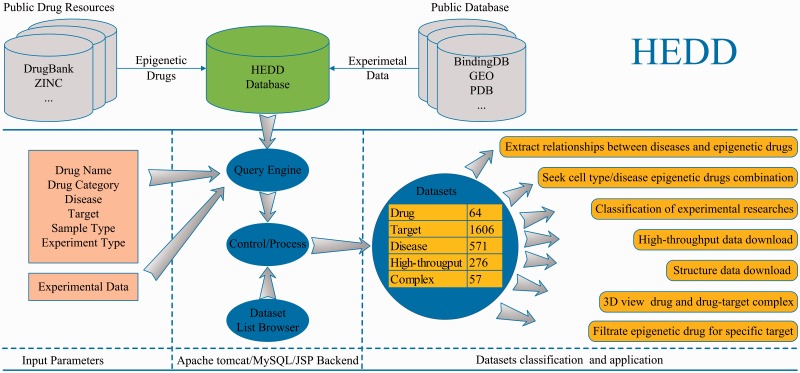
Overview of the establishment and workflow of HEDD. HEDD integrates epigenetic drugs and related experimental data from public drug resources and other public databases. Users can input multiple options to the query engine or use the dataset list browser to acquire epigenetic drugs and related datasets. Furthermore, this enables users to search epigenetic drugs for specific targets, and view the structure of drug and drug–target complex in 3D. All search results can be downloaded as flat format for further analysis.

HEDD was designed to store epigenetic drugs information for Homo species, which was created at 9/2016. The current version of HEDD consists of 64 epigenetic drugs collected from public drug resources: PubChem Compound, DrugBank and ZINC (29–31). Related experimental data (clinical trials, binding constants, high-throughput experimental data and co-crystal structure of the drug-target complex) were obtained from the following public databases: ClinicalTrials.gov, BindingDB, OMIM, GEO and PDB (34–38) (Table 2 and Figure 2). These experimental data are profiled by bio-assay (such as AlphaScreen assay), high-throughput assay (like gene expression array) and co-crystal with X-RAY diffraction.

**Table 2:** List of epigenetic drugs for the human epigenetic drug database

Drug Category	Epigenetic drugs
DNMTi	Decitabine, azacitidine, EGCG, zebularine, hydralazine, procainamide
HATi	C646, curcumin
HDACi	Vorinostat, givinostat, panobinostat, TSA, belinostat, entinostat, CG-1521, romidepsin, ITF-A, ITF-B, valproic acid, OSU-HDAC-44, HC-toxin, magnesium valproate, plitidepsin, tasquinimod, sodium butyrate, mocetinostat, carbamazepine, SB939, CHR-2845, CHR-3996, JNJ-26481585, sodium phenylbutyrate, pivanex, resveratrol, abexinostat, resminostat, dacinostat, droxinostat
HDMi	Pargyline, clorgyline, bizine, GSK2879552, GSK-J4, KDM5-C70, JIB-04, tranylcypromine
HMTi	EPZ-6438, GSK126, CPI360, DZNep, GSK343, EI1, BIX-01294, UNC0638, EPZ004777, UNC0224
PAHi	JQ1, CPI203, RVX-208, I-BET151, I-BET762, i-BET-726
PMHi	UNC669, UNC1215

The list is classified according to drug categories.

### Epigenetic drug relevant datasets

Before being finally stored, epigenetic drugs and related annotation information and experimental data were converted into five kinds of datasets in HEDD: (i) drug, (ii) target, (iii) disease, (vi) high-throughput and (v) complex. These 64 durg datasets provide the basic information and structure of epigentic drugs. Furthermore, the 1606 target datasets quantitatively describe the inhibitor action of epigenetic drugs to a specific target with four kinds of binding constants (Ki, IC50, Kd and EC50). In addition, the 571 disease datasets describe the application of epigenetic drugs for treating diseases. According to the period of research state, disease datasets are classified as follows: approved, in clinical trials, and preclinical. The 276 high-throughput datasets were probed from arrays, sequencing and RT-PCR to measure gene expression, SNP, genome binding/occupancy profiling and DNA melatyation profiles with the single or combination treatment of epigenetic drugs for Homo species. Furthermore, 57 complex datasets describe the co-crystal structure of the drug-target complex for epigenetic drugs using X-RAY diffraction or NMR.

### Functions provided by HEDD

Flexible query options and dataset list browsers are provided for the acquisition and investigation of epigenetic drugs and related datasets of interest (namely, drug, target, disease, high-throughput and complex). Users can specify the query options such as drug name and disease to acquire the specific dataset according to their own needs. They can also fast browse the dataset list according to dataset categories. The dataset pages provides information and experimental data of an epigenetic drug. Moreover, the structure of the drug and drug–target complex can be viewed through visualization modules based on the Jmol.php (https://chemapps.stolaf.edu/jmol/jmol.php); and structure and high-throughput data can be downloaded for further locally analysis.

## Database use and access

### Dataset list browser

**Figure 3:**
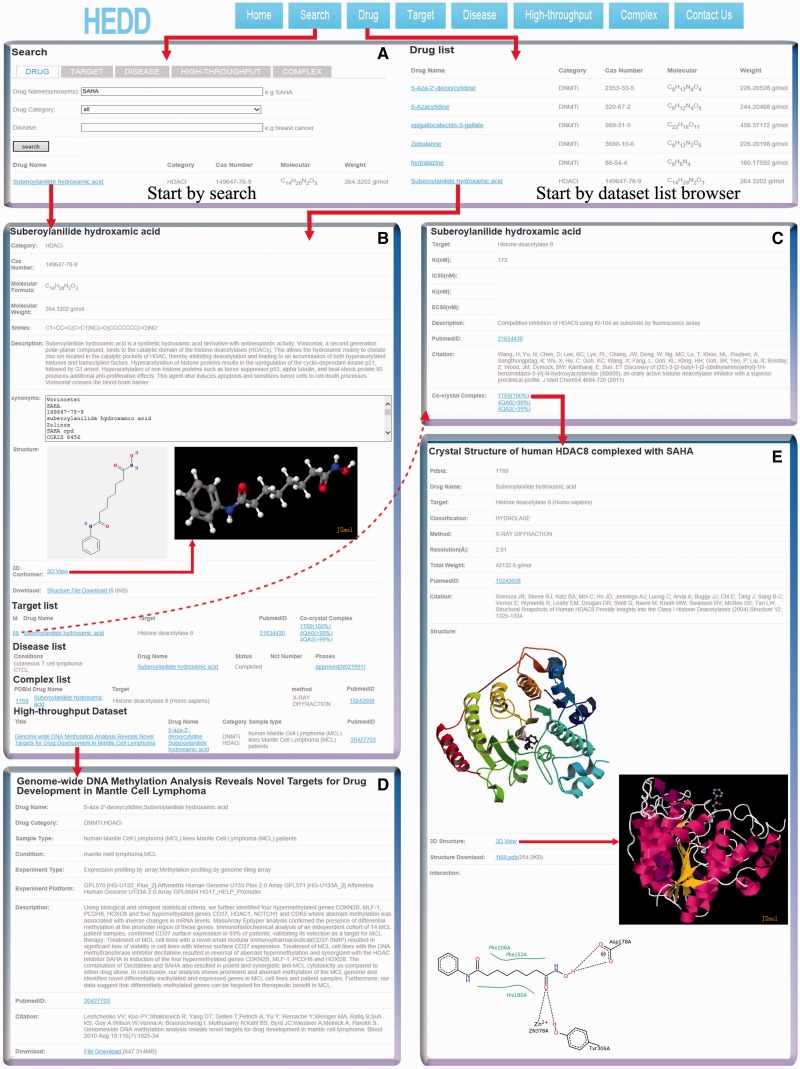
The screenshot shows the interrelation of search tools and datasets in HEDD. Users can start searching a dataset through the ‘Search’ menu or browse the dataset list to enter a dataset. (A) The screenshot showing the starting points of the search and dataset list browser. HEDD offers five advanced search options, namely, the search for drug, target, disease, high-throughput and complex datasets. This allows unlimited condition queries using drug names and diseases. (B) The screenshot shows the drug dataset of suberoylanilide hydroxamic acid. Users can obtain basic information of the drug, and are allowed to view its 3D structure and download the structure data. Related datasets of the drug are listed at the bottom of the page, which can be accessed through internal links. (C) The screenshot shows the target dataset. Users can obtain the quantitative description of a drug to a specific target. (D) The screenshot shows a high-throughput dataset. Users can download high-throughput data for local analysis. (D) The screenshot shows a complex dataset. Users can either download the structure data or view the structure of the drug–target complex in 3D.

HEDD is a highly modularized database, which facilitates data search and acquisition. An overview of HEDD and four result pages are shown in Figure 3. Figure 3A shows that starting points of the five dataset list browsers (namely, drug, target, disease, high-throughput, and complex). The dataset list browser was developed to allow the fast browsing of the datasets according to dataset categories. Taking the drug list browser as an example, by clicking the ‘Drug’ button, users can browse the basic information of an epigeneitc drug, including drug name, catogory, CAS number, molacular formula and weight. The dataset list browser exhibits 20 records each page and provides page turning. Users can enter the drug dataset page by clicking the drug name (blue icon), as shown in Figure 3A. The dataset list browsers of target, high-throughput and complex provide concise information of every kind of dataset. By browsing these information, users can decide whether to enter the corresponding dataset and download relevant experimental data. In addition, the browser page provides useful links, including Pubmed (with PubmedID) and drug datasets in HEDD (with drug name).

It is noteworthy to note the disease list browser. The disease list browser provides the drug name, condition (disease names or healthy), status (completed or terminated), NCT number (for in clinical trials) and phases. Links to the clinical trials database, ClinicalTrials.gov (34), are available with the ‘NCT number’. In the phases item, the NDA number is provided for approved epigenetic drugs with a hyperlink to the FDA, phase 0–4 for epigenetic drugs in clinical trials, and the label ‘preclinical study’ for preclinical epigenetic drugs with links to relevant high-throughput datasets.

### Using the search tool to retrieve datasets of the epigenetic drug

HEDD supports flexible queries for various epigenetic drugs and related datasets by providing the five search options (capital icons: DRUG, TARGET, DISEASE, HIGH-THROUGHPUT and COMPLEX), as shown in Figure 3A. Taking the DRUG search as an example, users can specify their query options such as drug name, category and disease, which is the most suggested option for new users of HEDD. If users are interested in a specific epigenetic drug, they should query by drug name (synonyms). For instance, users can select the option search DRUG and input ‘SAHA’ in the textbox labeled ‘Drug Name (synonyms):’, and click the ‘search’ button. The ‘suberoylanilide hydroxamic acid’ dataset is the result of this search, as shown in Figure 3A. In order to visually understand the search results, HEDD provides the same organizational form with the search results and dataset list browsers. Users can enter the corresponding dataset and download relevant experimental data.

DISEASE search is a module to display the application of epigenetic drugs in clinic. The user can separately or jointly use the drug name pull-down menu and the disease textbox to execute the disease search process. Using ‘suberoylanilide hydroxamic acid’ and ‘breast cancer’ as inputs, a report of five dataset summaries will be returned. TARGET search and COMPLEX search are two modules to study mechanistic details on the inhibitor action of epigenetic drugs. TARGET search focuses on quantitative descriptions and COMPLEX search focuses on explaining the interaction of spatial structures. Users can separately or jointly use the drug name pull-down menu and target textbox to execute the target search and complex search processes. Using ‘suberoylanilide hydroxamic acid’ and ‘histone deacetylase 8’ as inputs, results of the TARGET search and COMPLEX search would contain 62 and 4 datasets, respectively. HIGH-THROUGHPUT search is a specific module dedicated to study application of high-throughput experiments in epigenetic drugs researches. Users who are interested in gene expression, DNA methylation and histone modification pattern variations with treatment of epigenetic drugs for Homo species may find this module helpful. For instance, users interested in DNA methylation can select ‘5-Azacytidine’ or other options from the pull-down menu ‘Methylation profiling by array’, with others as default. In this case, a report of three high-throughput datasets annotated with 5-Azacytidine and DNA methylation summaries will be returned.

### Interpreting dataset pages with cases of suberoylanilide hydroxamic acid

HEDD contains five kinds of datasets. Users can acquire information and the experimental data of the epigenetic drug in certain dataset pages, according to their own needs. A snapshot of the suberoylanilide hydroxamic acid dataset is shown in Figure 3B. The page has three sections, namely, introduction, structure and dataset lists. The introduction section provides the basic information of the drug including drug category, CAS Number, synonyms names, molecular formula and weight, Smiles, and a brief description of the drug action. In order to study the structure of the drug, a panel of the structure data from PubChem Compound (29) was integrated into the drug dataset; which can be downloaded by clicking the button ‘Structure File Download’ (download format: .sdf). In the structure section, users can view 3D structures in the popup page of Jmol.php (with CAS number as the parameter), which allows the use of a mouse to accomplish the operation of a Jmol like spin, showing and hiding molecules, etc. The dataset list section provides a summary of related datasets of suberoylanilide hydroxamic acid. These datasets can be viewed by clicking the relevant links. A snapshot of the target dataset is shown in Figure 3C. The dataset was built based on the research of Wang *et al.* (39). In that research, they performed a series of 3-(1,2-disubstituted-1H-benzimidazol-5-yl)-N-hydroxyacrylamides that were designed and synthesized as HDAC inhibitors. The target dataset page provides the drug name (suberoylanilide hydroxamic acid), target name (histone deacetylase 8), binding constant (Ki:123 nM), a brief description, and a citation with the PubmedID.

A snapshot of the high-throughput dataset page is shown in Figure 3D. The dataset was built based on the research of Leshchenko *et al.* (40). Genome-wide DNA methylation analysis was used to illuminate novel targets in mantle cell lymphoma (MCL). Two epigenetic drugs, 5-aza-2'-deoxycytidine and suberoylanilide hydroxamic acid, were used in this study. Sample types are MCL cell lines and patients. Experiment types were expression profiling by array and methylation profiling by genome tiling array. Leshchenko and colleges found a prominent and aberrant promoter methylation in MCL, and suggested that differentially methylated genes could be targeted for therapeutic benefits in MCL. Users who want to analyze the high-throughput data locally can download the raw data by clicking the icon ‘File Download’, and the file size is labeled beside the icon. When the raw data is not available, the link to GEO is given.

A snapshot of the complex dataset is shown in Figure 3E. The page has two sections, namely, introduction and structure. The introduction section provides the title, PDBID, drug name, target name, classification, assay method, resolution, total weight and citation with PubmedID. The dataset was built based on the research of Somoza *et al.* (41). Using X-RAY diffraction, they described the first crystal structures of human HDAC8 made complex with four structurally diverse hydroxamate inhibitors. The structure also suggests how the phosphorylation of Ser39 affects HDAC8 activity. In order to study the structure of the drug-target complex, we integrated a panel of structure data from PDB (38) into the complex dataset, which can be downloaded (download format: .pdb). In the structure section, users can also view the 3D structure in the popup page of Jmol.php (with PDBID as the parameter). Moreover, schematism was adopted to interpret the interaction between drugs and targets within the binding pocket.

### System design and implementation

HEDD was developed using J2EE. Browser-based interfaces were built using JSP and Eclipse (Mars). HEDD is running on an Apache Tomcat web server (version 8.0) and a MySQL relational database (version 5.0). The server operating system is windows 2008 server R2 enterprise. Http://117.78.60.47 is the IP address of the HEDD website, which is equivalent to the domain name http://hedds.org. Jmol.php (version 1.1.5) was used for the 3D visual function. HEDD allows users to access all of the key features of the web application through their mobile device. FireFox explorer is recommended for using HEDD.

### Future perspective

The current release of the database is the first version of HEDD. It includes an abundant amount of experimental data of epigenetic drugs for Homo sapiens, which are useful for biologists and bioinformaticians. However, the experimental data and functionality of HEDD remains limited. With the aim of building a comprehensive drug database that focuses on epigenetic drugs, continued efforts would be made to update the HEDD data, add more data analysis tools and improve database functionality. In the future, the rapid profiling of high-throughput data, binding constants and co-crystal structure would allow more and more samples in Homo and other species to accumulate faster based on multi-experimental methods. We will continuously collect the latest datasets to keep HEDD up-to-date. Scientific community and researchers are encouraged to submit their experimental data on epigenetic drugs, in order to provide HEDD with the latest updates. To date, all data in HEDD were measured by experiments. Hence, the number of drug-target complexes in HEDD is much less than the number of targets datasets. Therefore, the data predicted in *in silico* approaches would be recorded into HEDD, such as molecular docking, virtual screening, pharmacophore modeling, molecular dynamics and similarity searching. As a resource to study the potential roles of epigenetic drugs in remodeling epigenetic modification, HEDD could be extended with utilities for the identification and confirmation of targets (genes and pathways) related to epigenetic drugs from large-scale high-throughput data (such as gene expression) (42, 43). Since mice are very important for modeling diseases and testing drugs, we would extend the research scope and integrate high-throughput data for mice treated with epigenetic drugs into HEDD. We expect that our continuous efforts would help develop and improve HEDD, contribute to the understanding of epigenetic mechanisms in disease development, and boost clinical application and the discovery of epigenetic drugs.
